# Supplementary material for: Collaborative intelligence in AI: Evaluating the performance of a council of AIs on the USMLE
Source: PLOS Digit Health. 2025 Oct 9;4(10):e0000787. doi: 10.1371/journal.pdig.0000787 (PMC12510544; doi:10.1371/journal.pdig.0000787)
Supplement: S2 File — (ZIP) [file pdig.0000787.s002.zip › S2 Data Raw data files containing unprocessed question inputs and LLM outputs including transcripts of deliberations/STEP3_Question-135-1_Type-MC-NJ.docx]

**Facilitator** **[Round 1]:** A 2-year-old boy is brought to the emergency department by his babysitter because of a 30-minute history of respiratory distress. The babysitter reports that she has cared for the patient on numerous occasions during the past year. The child's parents are currently out of town for business but will return later tonight. The babysitter says, ""He has had a runny nose since I started babysitting yesterday, but this afternoon he awoke from a nap with a barking sound and he was breathing real heavy."" She does not know the child's prior health history, nor does she know if the boy takes any medications on a regular basis. The child looks tired and sits upright on the babysitter's lap. He is obviously comfortable with his caregiver. Vital signs are temperature 38.7°C (101.7°F), pulse 110/min, respirations 28/min and labored, and blood pressure 85/50 mm Hg. Pulse oximetry on room air shows an oxygen saturation of 89%. Physical examination discloses inspiratory stridor. Neck is supple, tympanic membranes are normal, and there is a profuse nasal discharge. Examination of the pharynx discloses no abnormalities. Auscultation of the chest discloses equal air entry on both sides and no crackles. The remainder of the examination shows no abnormalities. Treatment with 40% oxygen via venturi mask and intravenous fluids is initiated. Despite continued supplemental oxygen and hydration, the child remains tachypneic and stridorous at rest. Oxygen saturation is now 93%. In addition to administering racemic epinephrine by nebulizer, which of the following is the most appropriate next step? (A) Intravenous aminophylline therapy (B) Intravenous antibiotic therapy (C) Intubation (D) Oral dexamethasone therapy (E) Rectal acetaminophen therapy

**AI_A:** RESTATING THE QUESTION: This query revolves around determining the best next treatment step for a 2-year-old boy showcasing symptoms of respiratory distress. The symptoms described include a runny nose, heavy breathing, and a barking sound, paired with physical observations of inspiratory stridor and profuse nasal discharge. Despite an oxygen and hydration-based treatment approach, the boy remains stridorous and tachypneic while at rest, with oxygen saturation improving from 89% to 93%. PLANNING: The base question assumes the boy's symptoms are indicative of the underlying illness. That said, it's crucial to consider alternate assumptions. For instance, might these symptom presentations manifest from a different primary ailment? Or perhaps these symptoms, in conjunction with the patient's age and presenting conditions, suggest a particular disease process? Identifying a clinical priority within these assumptions will assist the treatment decision. CLINICAL REASONING: The reported symptoms of a "barking" cough, heavy breathing, stridor, and non-remitting respiratory distress, despite oxygen therapy, strongly point towards croup, also known as laryngotracheobronchitis. This is common in children aged 6 months to three years and is characterized by the inflammation and swelling of the larynx, trachea, and bronchial tubes. This inflammation can cause airway obstruction leading to the aforementioned symptoms. The management of croup involves administering nebulized epinephrine to provide temporary relief and steroids to reduce inflammation. Given that the patient's respiratory distress remains unabated despite initial management, introducing a steroid seems like a beneficial next step to reduce airway inflammation and improve symptoms. CONCLUDING: According to the clinical thought process and the specifics of this clinical scenario, these symptoms and their persistence despite administered care suggest croup as a plausible diagnosis. Steroids, given their role in reducing inflammation—which provides symptom relief and eases airway obstruction—are the most likely next treatment steps for this patient. RESPONSE: Therefore, the most appropriate next step would be "(D) Oral dexamethasone therapy," given the suspected diagnosis of croup in this patient.

**AI_B:** 1) RESTATING THE QUESTION: This 2-year-old boy has been displaying symptoms of respiratory distress such as heavy breathing, and a barking sound while breathing. He has also had a runny nose recently. Despite the administration of supplemental oxygen and hydration, the child continues to face breathing difficulties. After administering a racemic epinephrine nebulizer, what should be the next step to alleviate the child's condition? "Aminophylline" is a drug that is used for relief and prevention of wheezing, shortness of breath, and chest tightness caused by asthma, chronic bronchitis, emphysema, and other lung diseases. "Dexamethasone" is a corticosteroid that's used to treat several conditions such as allergies, asthma, adrenal insufficiency, poison ivy, lupus, and certain types of arthritis. "Acetaminophen" is a medication used to treat pain and fever. It is typically used for mild to moderate pain relief. 2) PLANNING: This toddler's symptoms, which include a barking cough, labored breathing, and inspiratory stridor, strongly point towards a condition known as "croup." Given that the symptoms have not improved despite supplemental oxygen and hydration. The primary assumption is that the underlying cause is an inflammatory response in the upper respiratory tract, possibly caused by a viral infection. If so, the administration of corticosteroids, such as dexamethasone, would likely be the most suitable choice to reduce inflammation and ease breathing. 3) CLINICAL REASONING: Croup is often a viral disease and manifests in inspiratory stridor and a barking cough, both of which are present in the boy. Dexamethasone, a corticosteroid, is often effective at reducing inflammation associated with croup. Other options like aminophylline and rectal acetaminophen are less likely to be efficient because they're usually used for asthma and pain/fever relief, respectively. 4) CONCLUDING: To help manage his symptoms of croup (a viral upper respiratory tract infection), a dose of dexamethasone, an anti-inflammatory medication, would be beneficial. This should reduce inflammation, which in turn will help alleviate the stridor and respiratory distress. 5) RESPONSE: Hence, the most appropriate next step would be (D) Oral dexamethasone therapy.

**AI_E:** RESTATING THE QUESTION: The patient, a 2-year-old boy, has come to the emergency department experiencing symptoms of respiratory distress, including a recent history of a runny nose, heavy breathing, a barking cough sound, and inspiratory stridor, despite being provided supplemental oxygen and hydration treatments. He also shows other symptoms like tachypnea and low oxygen saturation levels. In addition to racemic epinephrine administration via a nebulizer, we are asked to identify the most appropriate next step from a list of options: intravenous aminophylline therapy, intravenous antibiotic therapy, intubation, oral dexamethasone therapy or rectal acetaminophen therapy. PLANNING: To answer this, we must first understand a few key medical terms. Inspiratory stridor is a harsh, vibrating sound heard during inspiration, often caused by upper respiratory obstruction. Tachypnea refers to rapid breathing, and supplemental oxygen and hydration are typical supportive treatments for respiratory distress. Racemic epinephrine is a bronchodilator used to ease airway obstruction. Now, the presentation of a barking cough, heavy breathing, and stridor indicates towards the diagnosis of croup, a common pediatric illness characterized by inflammation and swelling of the larynx and trachea. Other treatments for croup as mentioned in the options include aminophylline (a bronchodilator), antibiotics (for infections), intubation (for severe airway obstruction, should be avoided if possible), dexamethasone (a corticosteroid to reduce inflammation), and acetaminophen (for fever and discomfort). CLINICAL REASONING: Croup is often viral in nature, making antibiotic therapy (option B) less effective. Considering the patient's low oxygen levels, intravenous aminophylline therapy (option A) might not be effective enough as it is typically used for conditions like asthma, not croup. Rectal acetaminophen therapy (option E) may contribute towards reducing the fever but would not assist in alleviating the primary respiratory symptoms. Intubation (option C) is typically reserved for severe cases, and while this child's case is concerning, it hasn't been clearly indicated as being severe enough for such an invasive procedure yet. CONCLUDING: Comparatively, oral dexamethasone therapy (option D) can help in managing croup by reducing inflammation in the airways, improving symptoms, and potentially helping to avoid the need for hospitalization. This approach seems plausible given the child's symptoms and is commonly used in practice for moderate to severe croup. RESPONSE: Therefore, the most appropriate next step in this case would be (D) Oral dexamethasone therapy.

**AI_D:** RESTATING THE QUESTION: The presented scenario involves a 2-year-old boy experiencing respiratory distress characterized by labored breathing and a barking cough. The child also has a fever and a runny nose that started yesterday. He doesn't show any improvement in his respiratory symptoms despite treatment with oxygen and intravenous fluids; however, his oxygen saturation has slightly improved. The babysitter doesn't know any of the child's medical history including any regular medication he might be on. The next step in management is being questioned, with options including intravenous aminophylline therapy, intravenous antibiotic therapy, intubation, oral dexamethasone therapy, and rectal acetaminophen therapy. PLANNING: Before we dive into possible interventions, we should isolate the main symptoms and signs the child is displaying: a barking cough, respiratory distress, fever, and runny nose. These symptoms lead us to suspect Croup (or laryngotracheobronchitis), which is a common viral illness in pediatric patients, often caused by the parainfluenza virus. The classic presentation includes an upper respiratory tract infection (runny nose, fever) followed by a barking cough and stridor. Further supporting our assumption of Croup is the lack of pulmonary infiltrates on auscultation (as denoted by no crackles) and stridorous breathing, indicative of upper airway obstruction. CLINICAL REASONING: Given these symptoms and the typical course of Croup, it's important to approach treatment in a stepwise fashion. Mild cases can typically be managed with supportive measures, including humidified air and hydration. However, if a patient such as this child shows no improvement or has more severe symptoms such as increased work of breathing and stridor, next steps would include administering nebulized epinephrine and corticosteroids. Nebulized epinephrine can afford quick, short-term relief by reducing upper airway swelling through vasoconstriction, while corticosteroids help reduce inflammation and edema in the upper airway over a longer period. The question specifies that racemic epinephrine has already been administered and asks about the next step, which suggests we should be looking at the options for longer-term management. Antibiotics would not generally be effective given Croup's viral origin, while aminophylline and acetaminophen don't address the inflammation and edema causing the symptoms. CONCLUDING: Given the child's persistent symptoms, the most appropriate next step would not be another fast-acting treatment, the administration of a medication not typically used for this condition, or an invasive procedure like intubation, which we might consider if the child had life-threatening symptoms. Instead, we would likely want to administer corticosteroids to address the ongoing inflammation and, hopefully, cut down the duration of symptoms. RESPONSE: So, the most appropriate next step in this scenario would be (D) Oral dexamethasone therapy.

**AI_C:** RESTATING THE QUESTION: A two-year-old boy is in the emergency room with symptoms of respiratory distress, a recent runny nose, a barking sound during breathing, and labored respirations. He has no known medication use or past health problems. Despite oxygen supplementation and hydration management, the boy remains tachypneic and stridorous. His physical examination, vitals, and pulse oximetry readings raise concerns. A question arises what the next appropriate step would be, after the administration of racemic epinephrine, amongst given options which include Intravenous aminophylline therapy, antibiotics intravenously, intubation, or oral dexamethasone, or rectal acetaminophen. PLANNING: This scenario presents a case of respiratory distress in a child with symptoms of heavy breathing, a barking cough, and jogfulness to keep an upright posture. These are suggestive of croup or laryngotracheobronchitis, typically caused by Parainfluenza virus and characterized by subglottic edema leading to narrowing of the airway. The major assumptions we must address are whether this condition is correctly diagnosed and whether the choice of treatment aligns with this diagnosis. CLINICAL REASONING: Treatment of croup includes nebulized racemic epinephrine, which has already been given as per the case. Racemic epinephrine acts by stimulating alpha-adrenergic receptors in the respiratory mucosa, causing vasoconstriction and a decrease in airway edema. Despite this, his observable distress continues pointing towards the severity of his condition. IV treatments like aminophylline are primarily used for conditions like bronchial asthma, while IV antibiotics don't fit the typical viral etiology of croup. On the other hand, dexamethasone, a corticosteroid, can help reduce airway swelling in croup and improve symptoms like stridor, typically within six hours of administration. It could also potentially prevent intubation, which is indicated only when the patient does not respond to other treatments, is rapidly deteriorating, or has already developed complications like complete airway obstruction or respiratory failure. Although acetaminophen can reduce fever, it would not address the primary pathology driving the child's symptoms. CONCLUDING: Considering the presented symptoms, likely diagnosis, the severity of the symptoms, woman-to-man transmission of STIs, current treatments given, and plausible treatment options, the next course of action should align with the standard of care principles. RESPONSE: Therefore, the most suitable next step to manage this child's respiratory distress, considering both symptom management and addressing the underlying condition, would be (D) Oral dexamethasone therapy.

**Facilitator** The consensus statement synthesizes the responses of AI_A, AI_B, AI_C, AI_D, and AI_E as follows: The question revolves around a 2-year-old boy presenting with symptoms of respiratory distress, including a runny nose, heavy breathing, a barking cough, and inspiratory stridor. Despite receiving supplemental oxygen and hydration, his symptoms persist. The boy's symptoms strongly suggest a diagnosis of croup, also known as laryngotracheobronchitis, a common condition in children of his age group. Croup is characterized by inflammation and swelling of the larynx, trachea, and bronchial tubes, leading to airway obstruction and the observed symptoms. The boy's persistent respiratory distress, despite initial management, indicates the need for further treatment. Among the options provided, intravenous aminophylline therapy, intravenous antibiotic therapy, intubation, oral dexamethasone therapy, and rectal acetaminophen therapy, the most suitable next step appears to be oral dexamethasone therapy. Aminophylline is typically used for conditions like asthma and may not be effective enough in this case. Antibiotics are less likely to be effective given croup's viral origin. Intubation is a more invasive procedure and is usually reserved for severe cases or when the patient does not respond to other treatments. Although acetaminophen can reduce fever, it would not address the primary pathology driving the child's symptoms. On the other hand, dexamethasone, a corticosteroid, can help reduce airway swelling in croup and improve symptoms like stridor, typically within six hours of administration. It could also potentially prevent the need for more invasive procedures like intubation. Therefore, the most appropriate next step in managing this child's respiratory distress, considering both symptom management and addressing the underlying condition, would be oral dexamethasone therapy.
